# A Gross Misapprehension

**Published:** 1884-11

**Authors:** 


### A GROSS MISAPPREHENSION.

Dr. Catching of the *Southern Dental Journal* seems to have quite the advantage of Brother Barrett, of the INDEPENDENT PRACTITIONER, on the subject of veracity. We don't mean to say that any one has intentionally lied; but brother B. will undoubtedly be very cautious hereafter in charging anything of the kind (*what kind?*) on Dr. Catching, and if he does (*does what?*), and the documents are sent to him to disprove the misstatement, we think Dr. B. will prefer acknowledging his mistake rather than to have the proof in detail published by a rival journal.—*Items of Interest.*

This beautiful specimen of hashed English is what comes from comprehending with one's elbows. There is no question of veracity whatever between Dr. Catching and "Brother Barrett," nor is there, so far as the latter knows, any misunderstanding between them. Prof. Abbott asserted that he never signed his name, nor authorized it to be signed, to a certain announcement. The correspondence published conclusively proves that he was undeniably right. If the editor of *Items of Interest* cannot or will not see that there is a vast difference between approving a proposed object in a private letter, and publicly committing one's self to a definite time, place, and plan for its accomplishment, it must be due to a phenomenal mental obtuseness, or—to something worse. The INDEPENDENT PRACTITIONER does not publish private letters without the consent of their writers. That consent was, in this case, for good and valid reasons, no doubt, refused, and the editorial pages were offered to Dr. Catching for the purpose of making any explanation, not incompatible with the truth, that should be satisfactory to him. This he declined, and as we could not print the private correspondence of gentlemen without due authorization, the matter was left for him to dispose of as he thought proper, and had not the misstatements of the editor of *Items of Interest* made it evident that at least one person had mentally fumbled the whole affair, it would not again have been alluded to by us.

